# Histopathological Remodeling and Reduced Hypoxia-Related Marker Expression Following Marsupialization of Ameloblastoma: A Paired Analysis

**DOI:** 10.3390/biomedicines14102336

**Published:** 2026-10-14

**Authors:** Shuichi Ishida, Kiyofumi Takabatake, Takuma Arashima, Hiroshi Funahashi, Masae Fujii, Hotaka Kawai, Keisuke Nakano, Katsuhiko Amano, Seiji Iida, Hitoshi Nagatsuka

**Affiliations:** 1 Department of Oral and Maxillofacial Reconstructive Surgery, Graduate School of Medicine, Dentistry and Pharmaceutical Sciences, Okayama University, 2-5-1 Shikata-cho, Kita-ku, Okayama 700-8525, Japan; de428003@s.okayama-u.ac.jp (S.I.); ppki7efq@s.okayama-u.ac.jp (H.F.); 2 Department of Oral Pathology and Medicine, Faculty of Medicine, Dentistry and Pharmaceutical Sciences, Okayama University, 2-5-1 Shikata-cho, Kita-ku, Okayama 700-8525, Japan; de18018@s.okayama-u.ac.jp (H.K.); pir19btp@okayama-u.ac.jp (K.N.); jin@okayama-u.ac.jp (H.N.); 3 Department of Oral Pathology and Medicine, Graduate School of Medicine, Dentistry and Pharmaceutical Sciences, Okayama University, 2-5-1 Shikata-cho, Kita-ku, Okayama 700-8525, Japan; de428001@s.okayama-u.ac.jp (T.A.); gmd20089@s.okayama-u.ac.jp (M.F.); 4 Department of Oral and Maxillofacial Reconstructive Surgery, Faculty of Medicine, Dentistry and Pharmaceutical Sciences, Okayama University, 2-5-1 Shikata-cho, Kita-ku, Okayama 700-8525, Japan; amanokatsuhiko@okayama-u.ac.jp; 5 Department of Dental Hygiene, Kyoto Koka Women’s College, 38 Nishikyogokukadono-cho, Ukyo-ku, Kyoto 615-0882, Japan; s-iida@mail.koka.ac.jp

**Keywords:** ameloblastoma, marsupialization, hypoxia, HIF-1α, tumor microenvironment

## Abstract

**Background:** Marsupialization is widely used as a jaw-preserving treatment for large cystic ameloblastomas and is generally believed to reduce tumor volume through decompression. However, the biological mechanisms underlying tumor regression remain poorly understood. This study aimed to investigate the histopathological and hypoxia-related molecular changes in ameloblastoma before and after marsupialization using paired specimens from the same patients. **Methods:** This retrospective paired study included 13 patients with ameloblastoma who underwent marsupialization (median duration, 3 months; range, 1–12 months) followed by enucleation with peripheral ostectomy. Histopathological and immunohistochemical changes were compared between paired pre- and post-marsupialization specimens obtained from the same patients. Expression of the hypoxia-related molecules HIF-1α, carbonic anhydrase IX (CA IX), glucose transporter 1 (GLUT1), vascular endothelial growth factor (VEGF), and the proliferation marker Ki-67 was evaluated. **Results:** Marsupialization was associated with characteristic histopathological alterations, including increased squamous metaplasia, reduced stellate reticulum-like morphology, and a shift from myxoid to fibrous stromal patterns. Nuclear HIF-1α expression decreased significantly after marsupialization, accompanied by coordinated reductions in CA IX, GLUT1, VEGF, and the Ki-67 labeling index. These findings were consistent with coordinated attenuation of hypoxia-associated signaling together with reduced Ki-67 labeling. **Conclusions:** This study provides within-patient pathological evidence of histopathological remodeling and reduced hypoxia-related marker expression following marsupialization in ameloblastoma. These findings suggest that the tissue response to marsupialization may involve attenuation of hypoxia-associated signaling.

## 1. Introduction

Ameloblastoma is the most common benign odontogenic tumor and is characterized by locally aggressive behavior and a high recurrence rate despite its benign histological appearance [1,2,3,4]. A systematic review and meta-analysis estimated the pooled incidence of ameloblastoma at 0.92 cases per million person-years (95% confidence interval, 0.57–1.49) [5]. The same review reported a mean patient age of 34 years, with cases occurring most frequently in the third decade of life and a slight male predominance (53%) [5]. It most commonly arises in the posterior mandible and often remains asymptomatic until it reaches a considerable size, resulting in delayed diagnosis and extensive bone destruction [1,3]. Histologically, ameloblastoma is composed of epithelial islands resembling the enamel organ, with peripheral palisading columnar cells exhibiting reverse nuclear polarity and central stellate reticulum-like cells. Depending on the subtype, varying degrees of cystic degeneration and squamous metaplasia may also be observed [4,6].

Radical surgical resection with an adequate bony margin remains the standard treatment because of the tumor’s infiltrative growth pattern and propensity for local recurrence [1,2]. However, extensive resection frequently results in substantial functional and esthetic morbidity, particularly in younger patients. Marsupialization followed by definitive surgery has been reported for selected cystic ameloblastomas, particularly unicystic ameloblastoma, as well as selected conventional ameloblastomas with substantial cystic components [7]. This staged approach should be distinguished from the broader management of conventional ameloblastoma. Although marsupialization often induces gradual tumor shrinkage and new bone formation, the biological basis underlying this therapeutic response remains poorly understood.

Unlike odontogenic cysts, whose volume reduction can largely be explained by passive decompression and decreased intracystic pressure, ameloblastoma is a true epithelial neoplasm possessing intrinsic proliferative capacity. Biological changes in the tumor microenvironment may contribute to the response to marsupialization alongside mechanical decompression, although the relative contributions of these processes remain unclear.

Increasing evidence indicates that the biological behavior of ameloblastoma is regulated not only by the neoplastic epithelium but also by its surrounding microenvironment. Tumor growth is influenced by complex interactions involving stromal remodeling, extracellular matrix composition, angiogenesis, osteoclast activation, and multiple signaling pathways [8,9,10]. These findings support the concept that alterations in the tumor microenvironment may substantially modify the biological characteristics of ameloblastoma.

One of the key regulators of the tumor microenvironment is tissue hypoxia. Hypoxia profoundly influences tumor metabolism, proliferation, angiogenesis, invasion, and therapeutic response through activation of hypoxia-inducible factor-1α (HIF-1α) [11,12]. Under normoxic conditions, HIF-1α is rapidly degraded via the ubiquitin–proteasome pathway, whereas hypoxia stabilizes the protein, allowing its nuclear accumulation and transcriptional activation of numerous downstream genes involved in metabolic adaptation, angiogenesis, and cell survival. Among these downstream targets, carbonic anhydrase IX (CA IX), glucose transporter-1 (GLUT1), and vascular endothelial growth factor (VEGF) are well-established mediators of pH regulation, glucose metabolism, and angiogenesis, respectively [11,12].

Previous studies have demonstrated increased expression of HIF-1α and its downstream molecules in ameloblastoma, implicating hypoxia-related signaling in tumor progression and biological aggressiveness [13,14,15,16,17,18]. However, these investigations have been limited to untreated lesions and have provided only cross-sectional observations. Consequently, whether therapeutic intervention alters hypoxia-responsive signaling in ameloblastoma remains unknown.

Because marsupialization establishes communication between the lesion and the oral cavity while reducing intracystic pressure, it may also modify oxygen availability, vascular perfusion, and the overall tumor microenvironment. We hypothesized that these environmental alterations might attenuate HIF-1α-associated hypoxia-responsive signaling and contribute to reduced proliferative activity and histopathological remodeling.

To investigate this hypothesis, we conducted what is, to our knowledge, the first paired analysis of HIF-1α, CA IX, GLUT1, VEGF, and Ki-67 expression in ameloblastoma specimens obtained before and after marsupialization from the same patients. This unique study design minimizes interpatient variability and enables direct evaluation of treatment-associated biological alterations that cannot be assessed using conventional cross-sectional analyses. By comprehensively evaluating HIF-1α together with its downstream effectors CA IX, GLUT1, VEGF, and the proliferation marker Ki-67, we sought to characterize treatment-associated molecular and histopathological changes following marsupialization.

## 2. Materials and Methods

### 2.1. Patients and Tissue Samples

Medical records from 1 April 2005 to 1 April 2025 were retrospectively reviewed, and 26 patients with intraosseous ameloblastoma who initially presented to the Department of Oral and Maxillofacial Reconstructive Surgery at Okayama University Hospital were identified. Of these, 13 patients underwent marsupialization as a first-stage decompressive treatment followed by second-stage enucleation with peripheral ostectomy and were included in the paired analysis. The remaining 13 patients followed other treatment pathways, including resection, biopsy with wound closure followed by enucleation, or repeated marsupialization with serial tumor removal. Paired tissue specimens were available for all 13 included patients. Marsupialization was selected when a cystic cavity was confirmed at biopsy. In multilocular lesions, marsupialization was selected when substantial cystic components were present. In multilocular lesions, septa encountered during marsupialization were removed to establish communication between cystic compartments. Paired formalin-fixed, paraffin-embedded (FFPE) tissue specimens obtained from the incisional biopsy performed at the time of marsupialization and from the subsequent enucleation were retrieved from the archives of the Department of Oral Pathology. Because orientation information was unavailable for the post-marsupialization surgical specimens, anatomical correspondence with the initial biopsy site could not be determined.

The paired study design enabled direct comparison of histopathological and molecular alterations before and after treatment within the same patient, thereby minimizing interindividual variability. Clinicopathological data, including patient age, sex, tumor location, radiographic findings, and duration of marsupialization, were collected from the medical records.

Hematoxylin and eosin (H&E)-stained sections from paired pre- and post-marsupialization specimens were independently reviewed by two oral pathologists who were blinded to pre/post-treatment status and immunohistochemical findings. Stromal phenotype was classified by qualitative visual assessment of the myxoid and fibrous components across the entire evaluable section as myxoid (M), mixed with myxoid predominance (M > F), mixed with fibrous predominance (F > M), or fibrous (F). Squamous metaplasia was recorded as present when unequivocal squamous differentiation was identified within tumor nests. Disagreements were resolved through discussion and consensus, and the final consensus assessments are presented in Table 1. Histopathological classification was provisionally assigned on the basis of pretreatment incisional biopsy findings, using current WHO terminology [19]. Follicular and plexiform patterns and cystic architecture were recorded separately as morphological features.

The study was conducted in accordance with the Declaration of Helsinki and approved by the Ethics Committee of Okayama University Hospital (Approval No. 1703-042-001, 10 March 2017). Because of the retrospective nature of the study, the requirement for written informed consent was waived, and an opt-out approach was adopted in accordance with institutional guidelines.

### 2.2. Immunohistochemical Analysis

Immunohistochemistry was performed on 3-μm-thick formalin-fixed, paraffin-embedded tissue sections. After deparaffinization, endogenous peroxidase activity was blocked with hydrogen peroxide in methanol. Heat-induced antigen retrieval was performed using citrate buffer (pH 6.0) for HIF-1α, Ki-67, and VEGF, and Dako Target Retrieval Solution (Dako, Glostrup, Denmark; pH 9) for CA IX and GLUT1. Nonspecific binding was blocked with normal serum for HIF-1α, CA IX, VEGF, and Ki-67, whereas Protein Block Serum-Free (Dako) was used for GLUT1.

Sections were incubated overnight at 4 °C with antibodies against HIF-1α (clone EP1215Y, Abcam, Cambridge, UK; 1:200), CA IX (Proteintech, Rosemont, IL, USA; 1:200), GLUT1 (Proteintech; 1:300), VEGF (clone C-1, Santa Cruz Biotechnology, Dallas, TX, USA; 1:150), and Ki-67 (Dako; 1:50). HIF-1α, CA IX, VEGF, and Ki-67 were detected using the VECTASTAIN Elite ABC system (Vector Laboratories, Newark, CA, USA), whereas GLUT1 was detected using the EnVision+ System-HRP (Dako). Immunoreactivity was visualized with 3,3′-diaminobenzidine and counterstained with hematoxylin. Paired pre- and post-marsupialization specimens were stained under identical experimental conditions to minimize technical variability. Negative controls were prepared by omitting the primary antibody. The staining conditions for HIF-1α, VEGF, GLUT1, and Ki-67 were based on our previous experience with OSCC tissues. CA IX immunostaining was performed according to the manufacturer’s recommended protocol.

HIF-1α was evaluated as the principal hypoxia marker, whereas CA IX, GLUT1, and VEGF were examined as downstream molecules associated with pH regulation, glucose metabolism, and angiogenesis, respectively. Ki-67 was assessed as a marker of proliferative activity.

For quantitative analysis, areas of highest immunoreactivity (hot spots) were selected by the first author at ×20 magnification using the same criterion for all specimens, and the appropriateness of the selected areas was reviewed by an oral pathologist. Pre/post-treatment status was not used to guide field selection or counting; however, complete blinding could not be guaranteed because differences in specimen size and histomorphology could potentially indicate treatment status. Fields of identical area at ×20 magnification were used for all analyses. Five fields were evaluated for each marker in each specimen, except for CA IX in the post-marsupialization specimen from Case 3 and HIF-1α in the post-marsupialization specimen from Case 4, for which two evaluable fields were available. The reduced field counts reflected limited evaluable tumor tissue in the corresponding immunostained sections, and all evaluable fields were included in the analysis. The selected fields were analyzed using ImageJ software (version 1.54p, National Institutes of Health, Bethesda, MD, USA). For HIF-1α and Ki-67, thresholding in ImageJ was used to assist visual identification of immunopositive nuclei; nuclear segmentation and counting were not automated. Automated counting was not used because, in these specimens, intensity-based thresholding alone could not reliably distinguish tumor-cell nuclei from inflammatory and other non-neoplastic nuclei. Positive tumor-cell nuclei and total tumor-cell nuclei were manually counted using the Cell Counter function, and the nuclear-positive rate for each field was calculated as the number of positive tumor-cell nuclei divided by the total number of tumor-cell nuclei ×100. The specimen-level HIF-1α-positive rate and Ki-67 labeling index were calculated as the arithmetic mean of the field-specific percentages across all evaluable fields. The minimum numbers of tumor cells assessed per specimen across the evaluated fields were 387 for HIF-1α and 739 for Ki-67. Morphologically identifiable inflammatory cells were excluded from HIF-1α cell counts. For CA IX, GLUT1, and VEGF, quantitative assessment was restricted to tumor epithelial cells to allow a consistent cell-compartment-specific comparison of hypoxia-related marker expression. Stromal, endothelial, inflammatory, and non-neoplastic epithelial cells were excluded from the quantitative scores. Although VEGF immunoreactivity was also observed in stromal cells, stromal and vascular VEGF expression was not quantitatively assessed. The proportion of positive tumor cells was scored as 0 (<1%), 1 (≥1% to <31%), 2 (≥31% to <61%), or 3 (≥61%), and staining intensity was graded as 0 (negative), 1 (weak), 2 (moderate), or 3 (strong). Immunohistochemical scores were calculated by multiplying the proportion and intensity scores (range, 0–9), and the mean score from all evaluable fields was used for statistical analysis.

### 2.3. Statistical Analysis

Molecular pre/post comparisons used the two-tailed Wilcoxon matched-pairs signed-rank test, with the patient as the unit of analysis. Exact two-sided *p* values were calculated. Zero-difference pairs were excluded from the signed-rank calculations, and tied absolute differences were assigned average ranks. Differences in HIF-1α and Ki-67 labeling indices, as well as the immunohistochemical scores for CA IX, GLUT1, and VEGF, were analyzed. HIF-1α-positive rates and Ki-67 labeling indices were summarized using means. Because the composite IHC scores for CA IX, GLUT1, and VEGF were semiquantitative/ordinal measures, they were summarized using medians and interquartile ranges. The associations between marsupialization duration and paired changes in the HIF-1α-positive rate and Ki-67 labeling index (pre minus post, expressed in percentage points) were explored using Spearman’s rank correlation. Two-sided exact permutation *p* values were calculated for these correlations. Squamous metaplasia was assessed using the two-sided exact McNemar test. Stromal categories were ordered as M, M > F, F > M, and F (increasing fibrous content) and assessed using the two-sided exact sign test, comparing the numbers of changes toward more fibrous versus more myxoid categories and excluding unchanged pairs from that test. Both tests used exact binomial probabilities with a null probability of 0.5; no equal spacing between stromal categories was assumed. Molecular and correlation analyses were performed using GraphPad Prism version 11 (GraphPad Software, San Diego, CA, USA). The additional exact binomial tests and Holm–Bonferroni adjustments were calculated using Python version 3.12.14. To address multiplicity, the five molecular pre/post comparisons for HIF-1α, CA IX, GLUT1, VEGF, and Ki-67 were treated as one family, and the two exploratory histopathological comparisons as a separate family. Holm–Bonferroni adjustment was applied separately within each family. Both unadjusted and adjusted *p* values are reported, with adjusted *p* < 0.05 considered statistically significant. The two Spearman correlation analyses were exploratory, and their *p* values were not adjusted for multiplicity.

## 3. Results

### 3.1. Histopathological Alterations

The clinicopathological and histopathological characteristics of all cases are summarized in Table 1. Eight cases were provisionally classified as conventional ameloblastoma and five as unicystic ameloblastoma. The median duration of marsupialization was 3 months (interquartile range, 2–8.5 months; range, 1–12 months).

**Figure 1:**
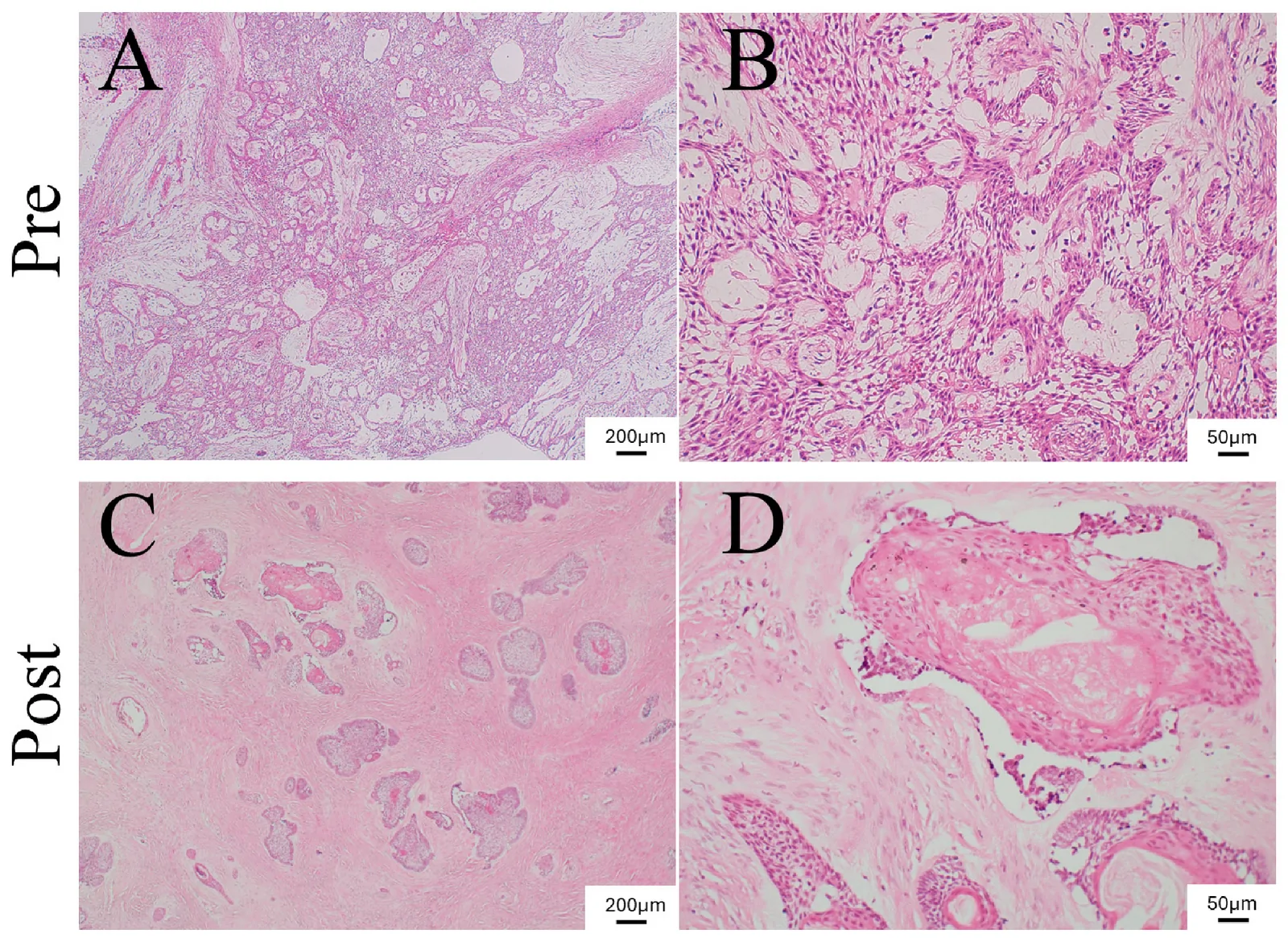
Histopathological findings before and after marsupialization. Pre-marsupialization sections at ×4 (**A**) and ×20 (**B**) show ameloblastomatous tumor nests composed of peripheral palisaded columnar or basaloid cells and central stellate reticulum-like cells within predominantly loose myxoid stroma. Post-marsupialization sections at ×4 (**C**) and ×20 (**D**) show prominent squamous metaplasia, reduced stellate reticulum-like morphology, cuboidal change of basal or peripheral tumor cells, and denser fibrous stroma. H&E staining.

Histopathological examination demonstrated several frequently observed morphological alterations following marsupialization (Figure 1A–D). Before treatment, ameloblastomas exhibited heterogeneous follicular and plexiform growth patterns composed of peripheral palisaded columnar or basaloid cells surrounding central stellate reticulum-like cells within a predominantly loose myxoid stroma (Figure 1A,B). Following marsupialization, squamous metaplasia became more prominent, the stellate reticulum-like component was reduced, and peripheral tumor cells frequently assumed a more cuboidal morphology (Figure 1C,D). Concomitantly, the stroma shifted from predominantly myxoid to denser fibrous patterns.

Squamous metaplasia was identified in 2 of 13 pre-marsupialization specimens and 8 of 13 post-marsupialization specimens. A shift toward a more fibrous stromal category was observed in 10 of 13 cases, and no post-marsupialization specimen retained a purely myxoid stromal pattern (Table 1). The stromal category was unchanged in Case 7 and shifted toward a more myxoid category in Cases 12 and 13. In the exploratory analyses, six cases changed from absent to present squamous metaplasia and none changed in the reverse direction (exact McNemar test, unadjusted *p* = 0.0313). For stroma, 10 versus 2 directional changes among 12 non-tied pairs yielded an unadjusted *p* = 0.0386 (exact sign test). Neither histopathological outcome was statistically significant after Holm–Bonferroni adjustment within the two-comparison family (both adjusted *p* = 0.0625).

### 3.2. Reduced HIF-1α Expression Following Marsupialization

**Figure 2:**
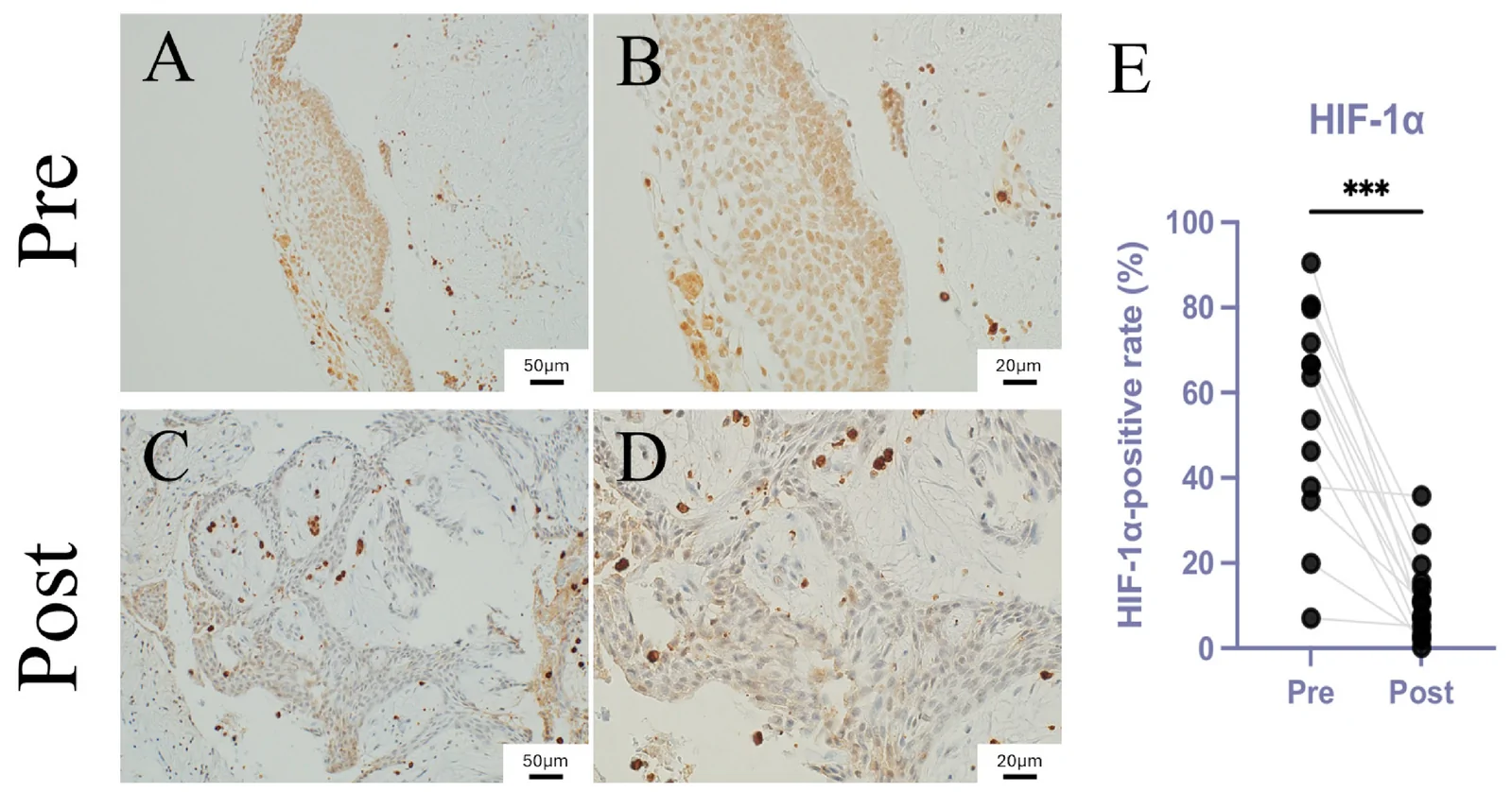
Representative HIF-1α immunohistochemical findings and paired quantitative analysis before and after marsupialization. Pre-marsupialization specimens are shown at ×20 (**A**) and ×40 (**B**), and post-marsupialization specimens at ×20 (**C**) and ×40 (**D**). Panel (**E**) shows paired nuclear HIF-1α-positive rates; lines connect specimens from the same patient. Asterisks denote unadjusted *p* values from two-sided exact Wilcoxon matched-pairs signed-rank tests: *** *p* ≤ 0.001. Multiplicity-adjusted interpretations across the five molecular comparisons are reported in Section 3.4.

Representative immunohistochemical findings and quantitative analysis of HIF-1α are shown in Figure 2A–E. Before marsupialization, strong nuclear HIF-1α expression was predominantly localized to peripheral tumor cells within ameloblastoma nests (Figure 2A,B). Following marsupialization, nuclear HIF-1α expression was markedly reduced (Figure 2C,D). HIF-1α-positive rates were significantly lower after marsupialization (mean, 55.3% before vs. 12.2% after; Figure 2E, unadjusted *p* < 0.001). Marsupialization duration was not significantly correlated with the decrease in the HIF-1α-positive rate (Spearman’s ρ = −0.031, *p* = 0.9232; *n* = 13).

### 3.3. Reduced Expression of HIF-1α Downstream Molecules

**Figure 3:**
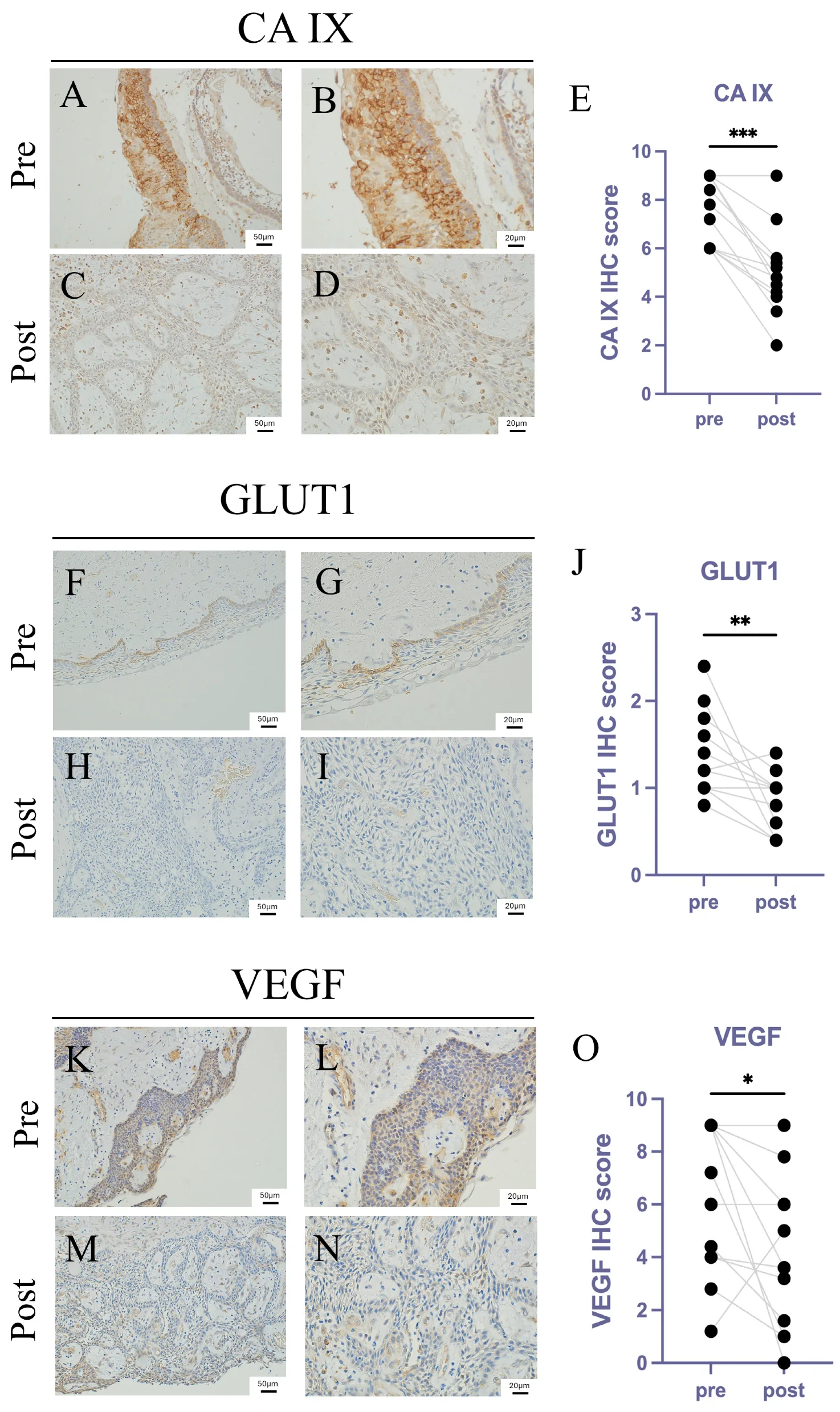
Representative immunohistochemical findings and paired quantitative analyses of CA IX, GLUT1, and VEGF before and after marsupialization. CA IX: pre ×20 (**A**), pre ×40 (**B**), post ×20 (**C**), post ×40 (**D**), and paired IHC scores (**E**). GLUT1: pre ×20 (**F**), pre ×40 (**G**), post ×20 (**H**), post ×40 (**I**), and paired IHC scores (**J**). VEGF: pre ×20 (**K**), pre ×40 (**L**), post ×20 (**M**), post ×40 (**N**), and paired IHC scores (**O**). Lines connect specimens from the same patient. Asterisks denote unadjusted *p* values from two-sided exact Wilcoxon matched-pairs signed-rank tests: * *p* < 0.05; ** *p* < 0.01; *** *p* ≤ 0.001. Multiplicity-adjusted interpretations across the five molecular comparisons are reported in Section 3.4.

Representative immunohistochemical findings and quantitative analyses of CA IX, GLUT1, and VEGF are shown in Figure 3. Before marsupialization, CA IX and GLUT1 immunoreactivity was predominantly localized to the cytoplasm of tumor epithelial cells, whereas VEGF expression was detected in both tumor cells and the surrounding stroma (Figure 3A–N). Quantitative analysis was restricted to tumor epithelial cells. Following marsupialization, immunoreactivity for all three markers was reduced. The median (interquartile range) immunohistochemical scores before and after marsupialization were 7.8 (6.0–9.0) and 4.8 (4.1–6.4), respectively, for CA IX (Figure 3E, unadjusted *p* = 0.0010); 1.2 (1.0–1.7) and 1.0 (0.4–1.0) for GLUT1 (Figure 3J, unadjusted *p* = 0.0039); and 7.2 (4.0–9.0) and 3.6 (1.3–6.9) for VEGF (Figure 3O, unadjusted *p* = 0.0234).

### 3.4. Reduced Ki-67 Labeling Index

**Figure 4:**
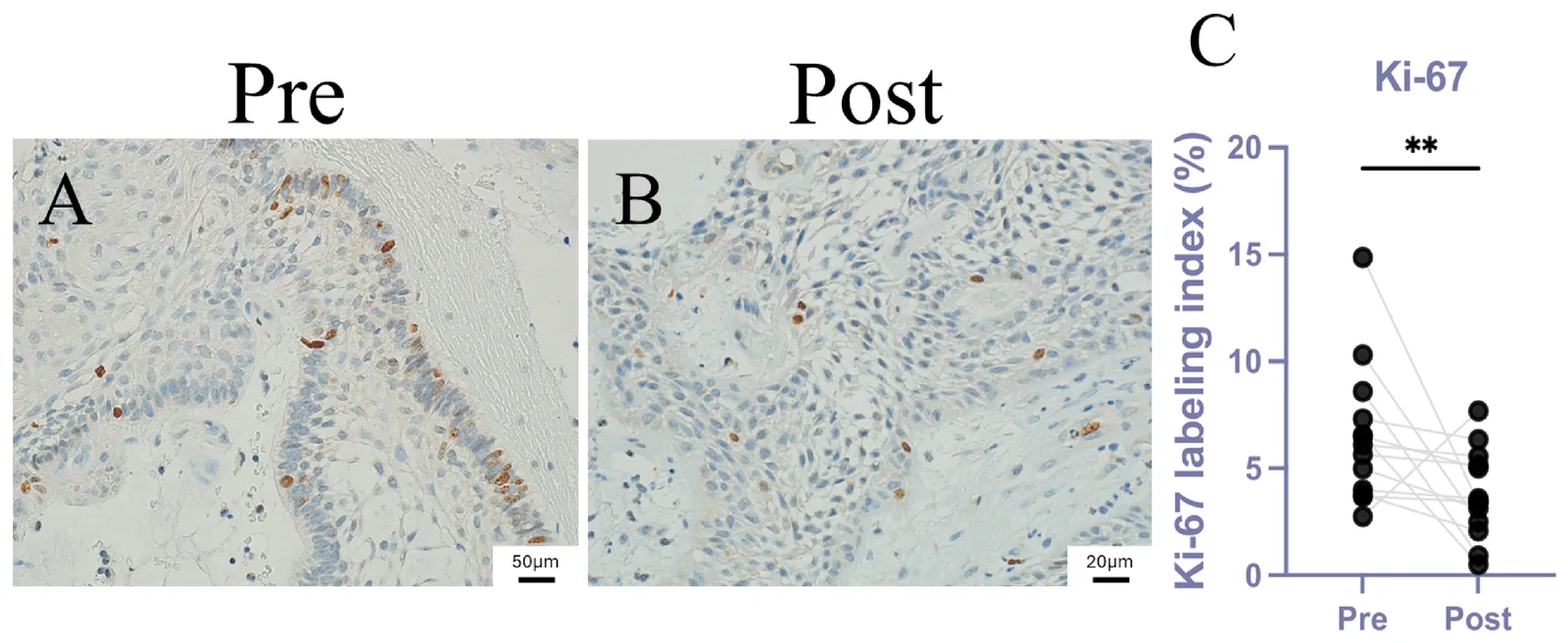
Representative Ki-67 immunohistochemical findings and paired quantitative analysis before and after marsupialization. Panels (**A**) and (**B**) show the pre- and post-marsupialization specimens, respectively, at ×40. Panel (**C**) shows paired Ki-67 labeling indices; lines connect specimens from the same patient. Asterisks denote unadjusted *p* values from two-sided exact Wilcoxon matched-pairs signed-rank tests: ** *p* < 0.01. Multiplicity-adjusted interpretations across the five molecular comparisons are reported in Section 3.4.

Representative immunohistochemical findings and quantitative analysis of Ki-67 are shown in Figure 4A–C. Before marsupialization, Ki-67-positive nuclei were predominantly observed in peripheral ameloblast-like cells (Figure 4A). Following marsupialization, the number of Ki-67-positive tumor cells was visibly reduced (Figure 4B). Ki-67 labeling indices were significantly lower after marsupialization (mean, 6.5% before vs. 3.8% after; Figure 4C, unadjusted *p* = 0.0081). Marsupialization duration was not significantly correlated with the paired change in the Ki-67 labeling index (Spearman’s ρ = −0.269, *p* = 0.3702; *n* = 13). All five molecular pre/post comparisons remained statistically significant after Holm–Bonferroni adjustment within the five-comparison family (all adjusted *p* < 0.05).

## 4. Discussion

The most important finding of the present study is that marsupialization was associated with coordinated histopathological and molecular alterations in paired ameloblastoma specimens obtained before and after treatment from the same patients. Rather than representing isolated changes in individual biomarkers, the concomitant reductions in nuclear HIF-1α expression, cytoplasmic immunoreactivity for CA IX, GLUT1, and VEGF, and the Ki-67 labeling index, together with characteristic stromal remodeling, are consistent with attenuation of a hypoxia-responsive marker profile accompanied by reduced Ki-67 labeling. To our knowledge, this is the first within-patient paired study to demonstrate these treatment-associated alterations. Our findings are consistent with treatment-associated remodeling of the tumor microenvironment.

Marsupialization is generally believed to reduce lesion volume by decreasing intracystic pressure. The biological basis underlying tumor regression following marsupialization remains incompletely understood. Because marsupialization establishes communication between the lesion and the oral cavity while relieving intracystic pressure, it may alter the intratumoral microenvironment, potentially through changes in oxygen diffusion and local tissue perfusion. Although tissue oxygenation was not directly measured in the present study, the coordinated reductions in HIF-1α, CA IX, GLUT1, and VEGF are consistent with the hypothesis that marsupialization may attenuate hypoxia-responsive signaling through modification of the local tumor microenvironment.

HIF-1α is a master regulator of the cellular response to hypoxia and orchestrates a broad transcriptional program involved in metabolic adaptation, angiogenesis, pH homeostasis, and cell survival [11,12]. Persistent activation of this pathway has been associated with aggressive biological behavior and therapeutic resistance in a variety of solid tumors [11]. In the present study, nuclear HIF-1α expression was significantly reduced following marsupialization, suggesting attenuation of hypoxia-responsive signaling within the tumor. More importantly, this reduction was accompanied by coordinated decreases in CA IX, GLUT1, and VEGF, indicating attenuation of an HIF-1α-associated hypoxia-responsive profile rather than isolated changes in individual biomarkers. These findings are consistent with treatment-associated changes in hypoxia-related marker expression and remodeling of the tumor microenvironment.

Previous immunohistochemical studies have consistently implicated hypoxia-related signaling in the biological behavior of ameloblastoma. Mendonça et al. reported increased expression of HIF-1α, VEGF, and VEGFR-2 in ameloblastoma compared with dental follicles and calcifying odontogenic cysts [13], whereas Arima et al. demonstrated expression of HIF-1α and CA IX, further supporting the involvement of hypoxia-associated pathways in tumor progression [14]. However, these studies were limited to untreated lesions and provided only cross-sectional observations. In contrast, the present study employed a unique within-patient paired design, enabling direct comparison of paired pre- and post-marsupialization specimens while minimizing interpatient heterogeneity. The coordinated reduction in HIF-1α and its downstream effectors observed in the present study therefore extends previous findings by showing treatment-associated changes in hypoxia-related marker expression.

CA IX, GLUT1, and VEGF are complementary downstream effectors of HIF-1α that mediate key adaptive responses to hypoxia, including regulation of intracellular pH, glucose metabolism, and angiogenesis, respectively [11,12,15,17]. Previous studies have demonstrated increased expression of these molecules in ameloblastoma, supporting the concept that hypoxia-responsive signaling contributes to its biological behavior [13,14,18]. In the present paired analysis, all three markers were significantly reduced following marsupialization, indicating that the observed molecular alterations extend beyond suppression of HIF-1α itself and involve coordinated attenuation of an HIF-1α-associated hypoxia-responsive profile.

The stromal alterations observed in the present study further support the concept that marsupialization is associated with changes in both the tumor epithelium and the surrounding tumor microenvironment. Previous studies from our institution have established that the stroma of ameloblastoma is biologically active rather than merely supportive. Sathi et al. demonstrated that myxoid stroma is associated with impaired bone formation and enhanced osteoclastogenic signaling [20], whereas subsequent studies identified sFRP-2 and CCN2 as important mediators of epithelial–stromal interactions that regulate tumor proliferation, stromal fibrosis, and bone remodeling [21,22]. Consistent with these observations, the present paired analysis demonstrated a shift toward a more fibrous stromal category in 10 of 13 cases after marsupialization, with no post-treatment specimen exhibiting a purely myxoid stromal pattern. These findings are consistent with treatment-associated stromal remodeling; however, the exploratory stromal and squamous-metaplasia analyses were not statistically significant after Holm–Bonferroni adjustment for the two histopathological comparisons. A recent systematic review of cannabinoids in oral inflammation and bone healing highlights potential links between inflammatory modulation and bone remodeling, although the evidence is heterogeneous and largely preclinical [23]. This provides broader biological context rather than direct evidence for the mechanisms of marsupialization in ameloblastoma.

Squamous metaplasia should also be interpreted within the context of the overall histopathological alterations observed after marsupialization. Although squamous metaplasia is a well-recognized feature of ameloblastoma and may occur independently of treatment [4,6], it should not be regarded as a surrogate marker of reduced tumor activity when considered in isolation. In the present paired analysis, however, increased squamous metaplasia was observed at the cohort level alongside coordinated reductions in HIF-1α, CA IX, GLUT1, VEGF, and Ki-67, together with a shift toward a more fibrous stromal phenotype. These findings indicate that squamous metaplasia is better interpreted as part of a coordinated treatment-associated remodeling process than as an isolated morphological hallmark of tumor regression. Altered epithelial differentiation, including squamous differentiation, and possible changes in inflammatory signaling should also be considered when interpreting the observed HIF-1α reduction, as inflammatory stimuli can induce HIF-1α under normoxic conditions in vitro [24]. Reduced mechanical pressure and changes in stromal composition may also contribute to the observed marker changes. Their contributions were not separately assessed in this study, and improved oxygenation remains a proposed mechanism for the observed reduction.

**Figure 5:**
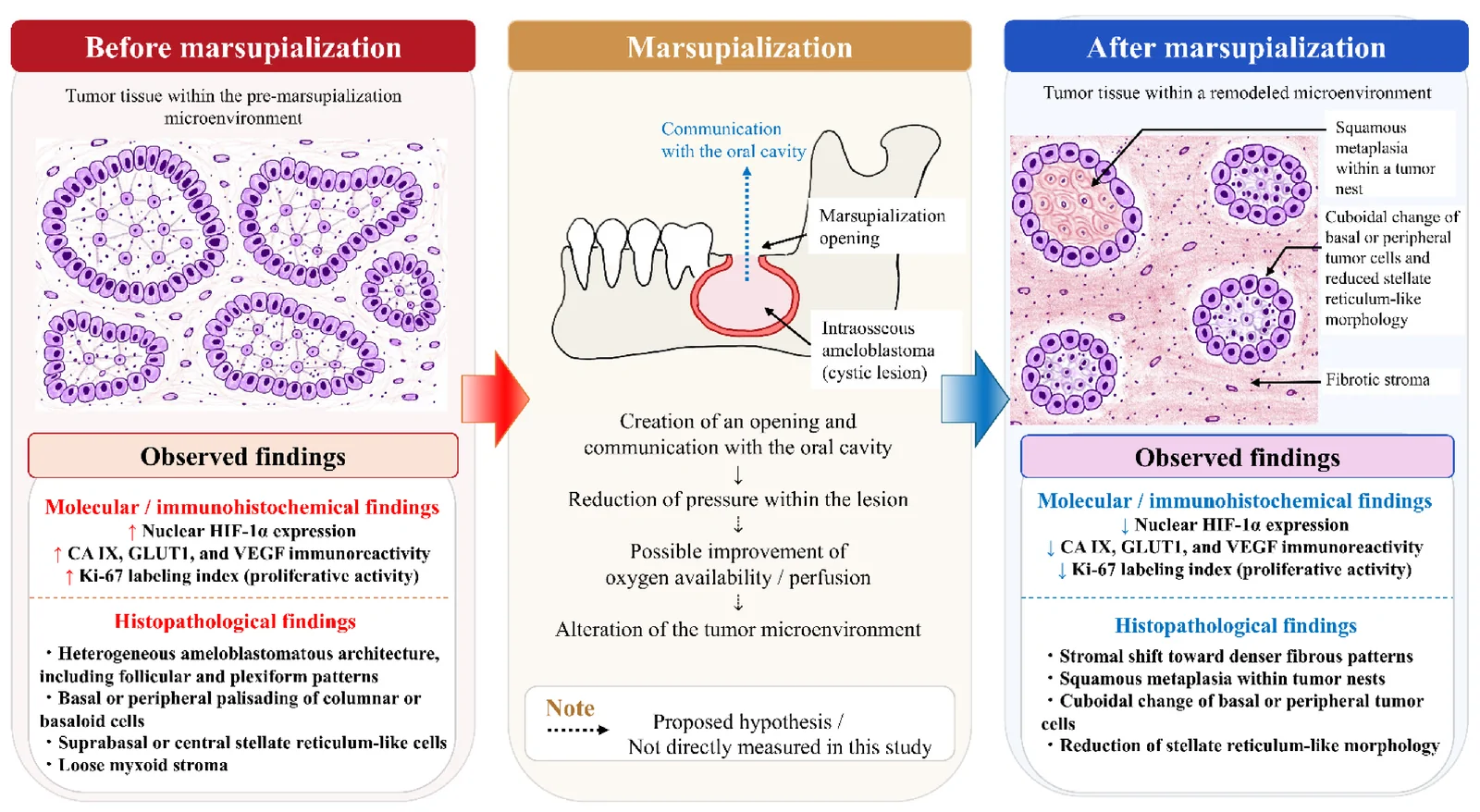
Schematic summary of the observed histopathological and molecular changes and the proposed microenvironmental mechanism associated with marsupialization. The left panel summarizes the pre-marsupialization state. Relative to post-marsupialization specimens, pre-marsupialization specimens showed greater nuclear HIF-1α expression, CA IX, GLUT1, and VEGF immunoreactivity, and Ki-67 labeling, together with heterogeneous ameloblastomatous architecture and predominantly loose myxoid stroma. The center panel shows creation of an opening and communication with the oral cavity, followed by reduction of pressure within the lesion. Changes in oxygen availability and local perfusion, and their causal links to the observed marker changes, are proposed mechanisms that were not directly assessed. The right panel summarizes the observed post-marsupialization changes, including reduced hypoxia-related marker expression and Ki-67 labeling, fibrous stromal remodeling, squamous metaplasia, cuboidal change of basal or peripheral tumor cells, and reduced stellate reticulum-like morphology. The red and blue solid arrows indicate the treatment sequence from the pre-marsupialization state to marsupialization and from marsupialization to the post-marsupialization state, respectively, whereas dashed arrows indicate proposed biological links.

Based on these findings, we propose a biological model in which marsupialization is associated with changes in hypoxia-related marker expression consistent with remodeling of the tumor microenvironment, together with reduced Ki-67 labeling, stromal remodeling, and increased squamous metaplasia. This proposed mechanism, which integrates the histopathological and molecular alterations identified in the present paired analysis, is summarized schematically in Figure 5.

From a clinical perspective, the present findings provide a potential biological explanation for the tissue changes observed following marsupialization in ameloblastoma. Traditionally, the clinical benefits of marsupialization have been attributed primarily to reduction of intracystic pressure and preservation of surrounding anatomical structures. A recent umbrella review reported lower recurrence rates after radical treatment, while evidence on postoperative morbidity and quality of life remained limited, emphasizing the need to balance recurrence control against treatment burden [25]. Evidence concerning staged marsupialization in selected cystic lesions, particularly unicystic ameloblastoma, should be distinguished from broader treatment-outcome evidence for conventional ameloblastoma [7,25]. The present findings suggest that the tissue response to marsupialization may also involve biological modulation of the tumor microenvironment associated with attenuation of an HIF-1α-associated hypoxia-responsive profile. However, this study was not designed to compare treatment strategies or assess recurrence, survival, functional outcomes, or optimal patient selection. These tissue findings alone do not justify broader indications for conservative treatment; treatment selection must consider recurrence risk, histopathological subtype, tumor location and extent, and patient age.

Several limitations of this study should be acknowledged. First, this was a retrospective study with a relatively small sample size, reflecting the rarity of paired pre- and post-marsupialization specimens available for analysis. Second, heterogeneity in histological subtype, lesion size, and duration of marsupialization may have influenced the observed histopathological and molecular changes. Although no significant correlations with marsupialization duration were detected, time-dependent effects on HIF-1α and Ki-67 expression cannot be excluded given the small sample size. Third, tissue oxygenation and vascular perfusion were not directly evaluated; therefore, the proposed mechanism linking marsupialization to attenuation of hypoxia-responsive signaling remains inferential. In addition, anatomical correspondence between the pre- and post-marsupialization sampling sites could not be confirmed, and spatial intratumoral heterogeneity may have contributed to the observed histopathological and immunohistochemical differences. Differences in specimen size between pretreatment biopsies and post-treatment surgical specimens may have affected hot-spot identification and the representation of heterogeneous tissue compartments, particularly for the semiquantitative assessment of CA IX, GLUT1, and VEGF. The within-patient paired design does not eliminate this sampling-related limitation. Interobserver agreement was not quantified, limiting assessment of the reproducibility of the histopathological evaluations. In addition, VEGF quantification was restricted to tumor epithelial cells; therefore, potential treatment-associated changes in stromal or vascular VEGF expression were not assessed. Finally, the present study relied primarily on histopathological and immunohistochemical analyses without complementary functional or molecular validation.

Despite these limitations, the within-patient paired study design represents a major strength of the present investigation, allowing direct evaluation of treatment-associated changes while minimizing the effects of interpatient biological variability. To our knowledge, this is the first study to demonstrate coordinated attenuation of an HIF-1α-associated hypoxia-responsive profile together with characteristic histopathological remodeling following marsupialization. Future prospective studies incorporating direct assessment of tissue oxygenation, comprehensive transcriptomic or proteomic analyses, and functional experimental models will be valuable for further elucidating the biological mechanisms underlying tumor regression after marsupialization.

## 5. Conclusions

The present paired study documents treatment-associated histopathological changes and reduced hypoxia-related marker expression in ameloblastoma following marsupialization. Attenuation of an HIF-1α-associated hypoxia-responsive profile, reflected by significant reductions in HIF-1α, CA IX, GLUT1, and VEGF, was accompanied by decreased Ki-67 labeling, stromal fibrosis, and increased squamous metaplasia. These findings are consistent with remodeling of the tumor microenvironment but do not establish improved oxygenation or HIF-1α-dependent pathway suppression as the underlying mechanism. Their clinical implications require validation in studies assessing treatment outcomes.

## Figures and Tables

**Table 1:** Clinicopathological and histopathological characteristics of the 13 ameloblastoma cases treated with marsupialization.

Case	Age (Years)	Sex	Anatomic Site	Radiographic Pattern	Duration of Marsupialization (Months)	Histopathological Subtype	Squamous Metaplasia, Pre → Post	Stromal Pattern, Pre → Post
1	29	Female	Left mandible, molar region	Unilocular	4	Conventional	Absent → Present	M → F > M
2	12	Male	Right mandible, molar–ramus region	Multilocular	1	Conventional	Absent → Present	M → F > M
3	16	Female	Right mandible, premolar–molar region	Multilocular	7	Unicystic	Absent → Present	M > F → F > M
4	24	Male	Right mandible, molar–ramus region	Predominantly unilocular	1	Unicystic	Absent → Present	M → F
5	66	Male	Left mandible, premolar region	Multilocular	3	Conventional	Absent → Absent	M → M > F
6	57	Female	Left mandible, premolar–molar region	Multilocular	2	Conventional	Present → Present	M > F → F > M
7	19	Female	Left mandible, molar–ramus region	Unilocular	12	Unicystic	Absent → Absent	F → F
8	35	Male	Left mandible, molar–ramus region	Unilocular	10	Conventional	Absent → Absent	M > F → F
9	12	Male	Right mandible, molar–ramus region	Unilocular	12	Unicystic	Absent → Absent	M > F → F
10	44	Male	Anterior mandible	Multilocular	3	Conventional	Absent → Absent	F > M → F
11	62	Female	Left mandible, molar region	Unilocular	2	Conventional	Absent → Present	F > M → F
12	19	Female	Right mandible, molar–ramus region	Unilocular	3	Unicystic	Present → Present	F > M → M > F
13	70	Male	Right mandible, molar–ramus region	Multilocular	7	Conventional	Absent → Present	F → F > M

Abbreviations: M, myxoid; M > F, mixed stroma with myxoid predominance; F > M, mixed stroma with fibrous predominance; F, fibrous. Squamous metaplasia was recorded as absent or present. Arrows indicate changes from pre- to post-marsupialization specimens. The classifications are provisional and based on pretreatment incisional biopsies. No definite mural proliferation was identified in the available biopsy sections of the five unicystic cases; its presence elsewhere in the lesion could not be reliably excluded.

## Data Availability

The raw data supporting the conclusions of this article will be made available by the authors on request.
